# Collodion baby

**DOI:** 10.11604/pamj.2021.40.77.26789

**Published:** 2021-10-06

**Authors:** Vinoth Kumar Perumal, Krishna Prasanth Baalann

**Affiliations:** 1 Department of Community Medicine, Sree Balaji Medical College and Hospital, Bharath Institute of Higher Education and Research, Tamil Nadu, India

**Keywords:** Collodion baby, gene mutation, ichthyosis

## Image in medicine

Lamellar ichthyosis or non-bullous congenital ichthyosis is an inherited autosomal recessive genodermatosis with a prevalence of 1 in 300,000 live births. The affected child is born with a tight, clear sheath covering their skin called a collodion membrane, that usually dries and peels off during the first few weeks of life, and then it becomes obvious that affected babies have scaly skin, and eyelids and lips that are turned outward. Infants with lamellar ichthyosis may develop infections, an excessive loss of fluids (dehydration), respiratory problems and even death within the first few days to weeks of life. Although the cause of this disorder is unknown, in some cases mutations in the Transglutaminase-1 (TGM1) gene are held accountable. We present to you a case of a term male baby born to a 31-year-old primigravid mother, with yellowish brown hard scales covering the whole body, but mostly the head, trunk, palm and soles with abnormally formed fingernails and toenails (nail dystrophy). The baby was examined for any other abnormalities. Skin biopsy showed epidermal hyperkeratosis and preservation of granular layer, confirming the diagnosis. The baby was treated in humidified environment with intravenous fluids and prophylactic antibiotics. Emollient was applied to the whole body. Rarity of such a case requires early and prompt diagnosis by clinicians, paying attention to skin care and hydration status of the baby, also mindful of any respiratory complications that may occur to prevent morbidity and mortality occurring from this condition.

**Figure 1:**
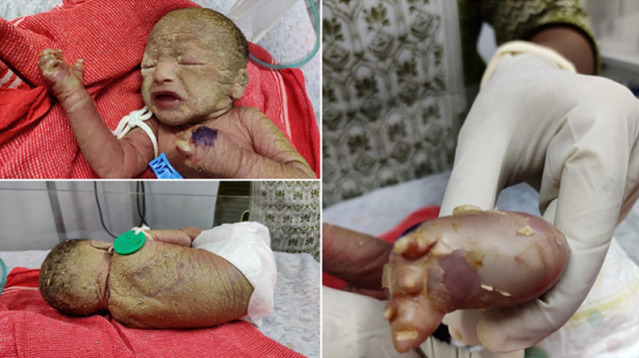
yellowish brown hard scales covering the whole body, the head, trunk, palm and soles with abnormally formed fingernails and toenails (nail dystrophy)

